# Supplementary material for: Telemedical communication patterns in myasthenia gravis in a remote monitoring study
Source: Neurol Res Pract. 2026 Sep 28;8(1):80. doi: 10.1186/s42466-026-00535-y (PMC13617854; doi:10.1186/s42466-026-00535-y)
Supplement: Supplementary file 1 [file 42466_2026_535_MOESM1_ESM.docx]

**Online Resource**

Article Title:

Telemedical communication patterns in myasthenia gravis in a remote monitoring study

Journal Name:

Neurological Research and Practice

Author Names and Affiliations:

Meret Herdick^1,2**^, Sophie Lehnerer, MD^1,2,3**^, Maximilian Mönch^4^, Lea Gerischer, MD^1,2^, Haoqi Sun^5^, Pushpa Narayanaswami^5^, Andreas Meisel, MD^1,2,6^, Maike Stein, MD^1,2,3,5*^

1 Charité – Universitätsmedizin Berlin, corporate member of Freie Universität Berlin and Humboldt-Universität zu Berlin, Department of Neurology with Experimental Neurology, Berlin, Germany

2 Charité – Universitätsmedizin Berlin, corporate member of Freie Universität Berlin and Humboldt-Universität zu Berlin, Neuroscience Clinical Research Center, Berlin, Germany

3 Berlin Institute of Health at Charité – Universitätsmedizin Berlin, Digital Health Center, Berlin, Germany

4 Charité – Universitätsmedizin Berlin, corporate member of Freie Universität Berlin and Humboldt-Universität zu Berlin, Institute of Biometry and Clinical Epidemiology, Berlin, Germany

5 Department of Neurology, Beth Israel Deaconess Medical Center/Harvard Medical, School, Boston, Massachusetts, USA

6 Charité – Universitätsmedizin Berlin, corporate member of Freie Universität Berlin and Humboldt-Universität zu Berlin, Center for Stroke Research Berlin, Berlin, Germany

* Correspondence:
Dr. Maike Stein, Charité – Universitätsmedizin Berlin, Department of Neurology with Experimental Neurology, Charitéplatz 1, 10117 Berlin, Germany, Email: [maike.stein@charite.de](mailto:maike.stein@charite.de)

 

***Online Resource 1*** *Association between age and the number of technical inquiries, with fitted linear trend and 95% confidence interval.*

***Online Resource 2*** *Topics addressed by specialists through the chat at different time points during study period*

|  | **Overall**  N=45 | | **Intervention group**  N=30 | **Control  group**  N=15 | **p-value\| Effect Size 95%-CI** |
| --- | --- | --- | --- | --- | --- |
| **Assessed during baseline visit, n (%)**  *“I have the feeling that I can reach a specialist in an adequate amount of time if my MG symptoms worsen.”* | | | | | 0.5^a^ \| 0.2^b^ (0.0 to 0.5) |
| Yes | 21 (46.7) | | 14 (46.7) | 7 (46.7) |  |
| Yes with limitations | 13 (28.9) | | 10 (33.3) | 3 (20.0) |  |
| No | 11 (24.4) | | 6 (20.0) | 5 (33.3) |  |
| **Assessed during end-of-study visit, 1 missing, n (%)**  *“I have the feeling that with MyaLink I can reach a specialist in an adequate amount of time if my MG symptoms worsen.”* | | | | |  |
| Yes | |  | 29 (100.0) |  |  |
| Yes with limitations | |  | 0 (0.0) |  |  |
| No | |  | 0 (0.0) |  |  |
| **p-value\| Effect Size** | |  | <0.1^c^ \| 0.5^d^ |  |  |
| ^a^ Pearson’s Chi-Squared test ^b^ Cramér’s V | | | | | |
| ^c^ Cochran’s Q test ^d^ Eta-squared (η^2^) | | | | | |
|  | | | | | |

***Online Resource 3*** *Availability of specialized medical services for the treatment of Myasthenia gravis*
